# Correction: Kos et al. An Experimental Study on the Properties of Concrete and Fiber-Reinforced Concrete in Rigid Pavements. *Materials* 2023, *16*, 5886

**DOI:** 10.3390/ma19091768

**Published:** 2026-04-27

**Authors:** Željko Kos, Sergii Kroviakov, Andrii Mishutin, Andrii Poltorapavlov

**Affiliations:** 1 Department of Civil Engineering, University North, University Centre of Varaždin, 104. Brigade 3, 42000 Varazdin, Croatia; 2 Department of Highways and Airfields, Odesa State Academy of Civil Engineering and Architecture, Didrichson Street 4, 65029 Odesa, Ukraine; skrovyakov@ukr.net (S.K.); mishutin52@ukr.net (A.M.); andpolt90@gmail.com (A.P.)

In the original publication [1], reference “Kroviakov, S.O.; Poltorapavlov, A.O.; Mishutin, A.V.; Zavoloka, M.V. The influence of the amount of fiber and superplasticizer on the strength of concrete for the rigid pavements. *Mod. Constr. Arch.* **2022**, *2*, 60–69. https://doi.org/10.31650/2786-6696-2022-2-60-69.” was not cited. The citation has now been inserted in Section Introduction, Paragraph 15, and should read:

“The development of fiber-reinforced concrete compositions for rigid pavement using local cement, aggregates, additives, and fiber is important for Ukraine, considering the significant amount of work that needs to be done to rebuild the infrastructure destroyed during the war. The current article is a further development of the previously published internal Ukrainian conference paper [27].”

With this correction, the order of some references has been adjusted accordingly.

The authors state that the scientific conclusions are unaffected. This correction was approved by the Academic Editor. The original publication has also been updated.
